# Acute Deterioration of Kidney Function after Total Hip Arthroplasty

**DOI:** 10.5704/MOJ.2007.020

**Published:** 2020-07

**Authors:** S Takeshita, M Sonohata, M Kitajima, S Kawano, S Eto, M Mawatari

**Affiliations:** Department of Orthopaedic Surgery, Saga University, Saga, Japan

**Keywords:** total hip arthroplasty, acute kidney injury, acute deterioration of kidney function, nonsteroidal anti-inflammatory drugs, diabetes mellitus

## Abstract

**Introduction:**

Post-operative acute kidney injury is a serious complication and identifying modifiable factors could assist in peri-operative management. This study aimed to identify the pre-operative and intra-operative factors associated with the incidence of post-operative acute kidney injury and acute deterioration of kidney function after total hip arthroplasty.

**Materials and methods:** This single-center, retrospective, observational study included 203 patients who underwent unilateral primary total hip arthroplasty. Acute kidney injury was determined using biochemical markers according to the risk, injury, failure, loss of kidney function, and end-stage kidney disease (RIFLE) criteria. Acute deterioration of kidney function was defined as the reduction of estimated glomerular filtration rate by ≥10ml/min/1.73m^2^.

**Results:**

Prior to total hip arthroplasty, 20% of all patients met the chronic renal dysfunction criterion of glomerular filtration rates <60ml/min/1.73m^2^ (glomerular filtration rate categories G3a-G5). Incidence rates of acute kidney injury and acute deterioration of kidney function after total hip arthroplasty were 0.49% and 6.9%, respectively. Multivariate regression analysis showed that diabetes mellitus and use of nonsteroidal anti-inflammatory drugs before total hip arthroplasty were significant risk factors for acute deterioration of kidney function. Advanced age, preoperative renal dysfunction, antihypertensive, diuretics, or statin use, operation time, total blood loss, type of anesthetic, and body mass index were not significant risk factors.

**Conclusion:**

Diabetes mellitus and use of nonsteroidal anti-inflammatory drugs were controllable risks, and multidisciplinary approaches are a reasonable means of minimising peri-operative acute kidney injury or acute deterioration of kidney function.

## Introduction

Total hip arthroplasty (THA) is a successful procedure that improves the quality of life and function of patients with hip osteoarthritis^1^. Therefore, THA is commonly performed for hip osteoarthritis, and the demand for primary and revision arthroplasties is expected to increase exponentially with time^2^. Although THA is considered a safe elective surgery, it may be associated with serious complications such as aseptic loosening and infections^3^. Moreover, the surgical stress of total joint arthroplasty may be too great for patients.

Post-operative acute kidney injury (AKI) is a serious complication and identifying modifiable factors could assist in peri-operative management^4^. AKI is a condition in which the renal function rapidly declines and becomes deficient, resulting in inability to maintain body fluid homeostasis^5^. AKI after cardiac surgeries has been investigated by many studies^6-8^, but AKI after total joint arthroplasty, particularly after THA, has rarely been evaluated^9-16^. The incidence of AKI after THA ranges from 0.19 to 8.1%^10-12, 15^. A previous systematic review and meta-analysis of acute kidney injury following total hip arthroplasty^17^ examined seventeen cohort studies that enrolled a total of 24,158 patients undergoing THA. The report determined that the overall estimated incidence rates of AKI and severe AKI requiring dialysis in patients undergoing THA were 6.3% and 0.5%, respectively. Furthermore, this study showed found that the incidence of AKI following THA was 9.2% in Asia, 8.1% in Australia, 7.4% in Europe, and 2.8% in North America. In addition, it was shown that the incidence of AKI following THA in past reports varied, and differences in incidence according to study location were observed. In addition, previous studies used varying criteria for identification of AKI, which ultimately contributes to the unclear incidence of AKI after THA.

Prior studies reported that risk factors for AKI in patients undergoing THA included older age, higher body mass (BMI), reduced baseline estimated glomerular filtration rate (eGFR)/chronic kidney disease (CKD), diabetes mellitus (DM), nonsteroidal anti-inflammatory drugs (NSAIDs) use, and peri-operative blood transfusions^17^. However, risk factors for AKI in patients undergoing THA differ across reports and are not clearly elucidated. The incidence of AKI after THA in Japanese patients has not been previously reported. Therefore, in this study, we aimed to determine the incidence of post-operative AKI and acute deterioration of kidney function after THA in a Japanese population. We also aimed to identify the pre-operative and intra-operative factors associated with acute deterioration of kidney function after THA. Based on the factors identified, we suggest preventive measures to reduce AKI following THA.

## Materials and Methods

**Fig. 1:**
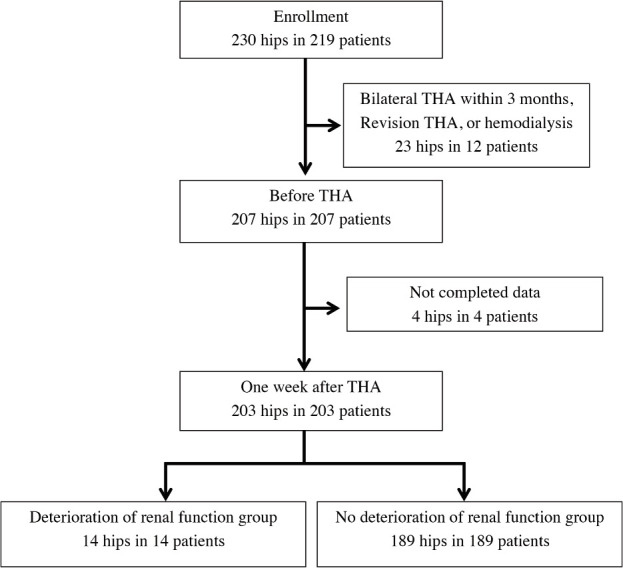
Flowchart of patient selection.

In this single-center, retrospective, observational study, THA of 230 hips in 219 patients was performed at our institution between January 2015 and June 2015. The indications for the procedure were severe hip pain and/or considerable difficulty in walking and performing daily activities. Patients who underwent bilateral THA within three months, revision THA, and hemodialysis, and those with incomplete data were excluded. Two hundred and three patients who underwent unilateral primary THA were finally included in the study (Fig. 1). The patients consisted of 34 men and 169 women, with an average age of 65.3 years (Table I).

**Table I:** Demographic Characteristics of the Study Participants

Number of patients	203
Number of hips	203
Male, female (number of patients)	34, 169
Age, years (mean±SD, range)	65.3±10.9, 37-90
Height, cm (mean±SD, range)	153±9.7, 118-179
Weight, kg (mean±SD, range)	57.3±11.8, 28.7-100
BMI, kg/m2 (mean±SD, range)	24.3±4.1, 16-43
Diagnosis (number of patients, (male, female))	
Developmental dysplasia of the hip	185 (25,160)
Osteonecrosis	7 (6,1)
Trauma	3 (2,1)
Rheumatoid arthritis	3 (0,3)
Rapidly destructive coxarthropathy	3 (0,3)
Perthes disease	2 (1,1)
Pre-operative NSAIDs use (number of patients, (%))	55 (27%)
Pre-operative ACEI/ARB use (number of patients, (%))	48 (24%)
Pre-operative diuretic use (number of patients, (%))	8 (3.9%)
Pre-operative statin use (number of patients, (%))	39 (19%)
History of diabetes mellitus (number of patients, (%))	20 (10%)
Anaesthetic type	
Spinal (number of patients, (%))	195 (96%)
General (number of patients, (%))	8 (4%)
Operation time (min)	49±19.0, 29-158
Total blood loss (g)	292±130.8, 14-704

*SD - standard deviation; BMI - body mass index; NSAIDs - nonsteroidal anti-inflammatory drugs; ACEI - angiotensin-converting enzyme inhibitor; ARB - angiotensin-II receptor blocker

All patients provided consent for use of their personal examination data in research studies. The study protocol adhered to the ethical guidelines of the 1975 Declaration of Helsinki, and the study was approved by the institutional review board.

All THAs were performed by four senior surgeons with the same cementless implants via a posterolateral approach. A suction drain was used and then removed two days after the operation. Patients followed the same clinical pathway, including standard post-operative care and use of prophylactic antithrombotics, analgesics, and prophylactic antibiotics. Cefazolin (1g) was intravenously administered once 30 minutes before THA and thrice after THA.

Patients received 15 or 30mg oral edoxaban once per day after THA for one week to prevent deep vein thrombosis. It has been reported that oral edoxaban 30mg once daily significantly reduced asymptomatic deep vein thrombosis after THA without increasing the incidence of bleeding complications^18^. Edoxaban is covered by Japan’s National Health Insurance for the prevention of venous thromboembolism after THA. Since the efficacy and safety of edoxaban treatment for 15 days has not been evaluated in Japan, the duration of treatment was set at 7 days in this study. Edoxaban dose was reduced or not used at all depending on age, weight, and renal function; 121 patients received 30mg; 63 patients received 15mg and 19 patients received none.

Laboratory data were obtained before and seven days after THA. Operative data, including operation time and total blood loss, were collected. Intra-operative blood loss was determined based on the contents of the suction bottle and the change in the weight of the surgical sponges used. Post-operative blood loss was determined based on the amount in the suction drain. In this study, we assessed total blood loss as a calculation of blood loss. To exclude the effects of peri-operative infusion, we assessed the total blood loss, which represents a direct measure of bleeding, rather than the change in hemoglobin levels.

Data on the medications used before THA, including angiotensin-converting enzyme inhibitors (ACEIs), angiotensin II receptor blocker (ARBs), diuretics, statins, antidiabetics, and NSAIDs, were obtained from the medication charts.

CKD before THA was determined according to an eGFR threshold of less than 60ml/min/1.73m^2,19^. AKI was determined using biochemical markers according to the RIFLE criteria^20^. The criteria were as follows: (i) risk: eGFR decrease >25% or 1.5 fold increase in serum creatinine or urine production of <0.5ml/kg/h for 6 h; (ii) injury: eGFR decrease >50% or doubling of creatinine or urine production <0.5ml/kg/h for 12 h; (iii) failure: eGFR decrease >75% or tripling of creatinine or creatinine >355μmol/l (with a rise of >44; >4mg/dl) or urine output below 0.3ml/kg/h for 24 h or anuria for 12 h; (iv) loss: persistent AKI or complete loss of kidney function for more than four weeks; and (v) end-stage renal disease: need for renal replacement therapy for > three months.

In this study, we defined the reduction of eGFR by ≥10ml/min/1.73m^2^ seven days after THA as “deterioration of renal function”. The number of AKI cases that met the RIFLE criteria after THA were few and would not allow definitive clarification of the associated risk factors. For this reason, we used separate criteria for cases with deterioration of renal function that did not meet the RIFLE criteria and examined the associated risk factors in these cases. We evaluated the incidence and risk factors for CKD and AKI at seven days after THA. Furthermore, we compared the “deterioration of the renal function group” and the “no deterioration of the renal function group”, and evaluated the risk factors for deterioration of renal function.

All statistical analyses were performed using SPSS for Windows software [version 23; IBM Corp, Armonk, NY]. The mean values of the two groups were compared using Student's t-test. Sex, treatment for DM, and pre-operative medication were compared using the chi-squared test. The correlation coefficient between age and eGFR before THA was analysed with Pearson's correlation analysis. A p-value <0.05 was considered statistically significant. Multivariate logistic regression was performed to determine those factors associated with post-operative acute deterioration of kidney function after THA and their odds ratios (ORs). The model included all variables with a p-value <0.30 in the univariate analysis.

## Results

**Fig. 2:**
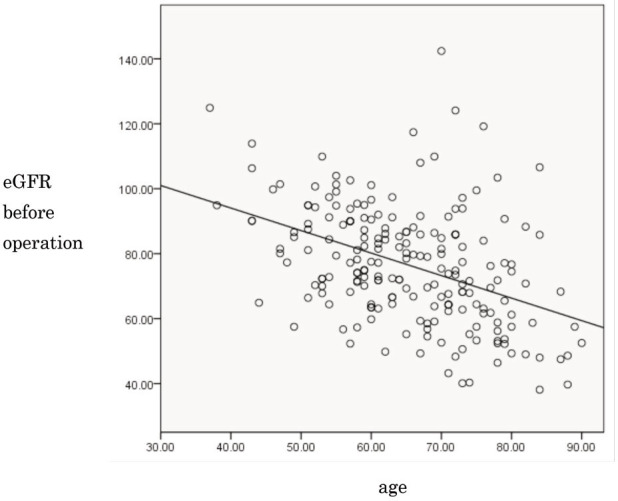
Relationship between age and pre-operative renal function (R=-0.420, p<0.001). eGFR, estimated glomerular filtration rate.

**Fig. 3:**
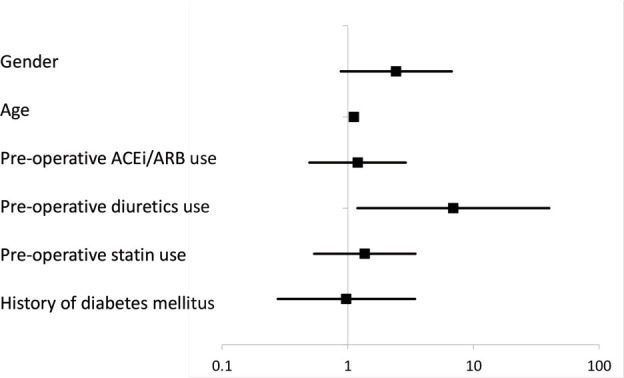
95% Confidence Interval of factors associated with preoperative CKD.

**Fig. 4:**
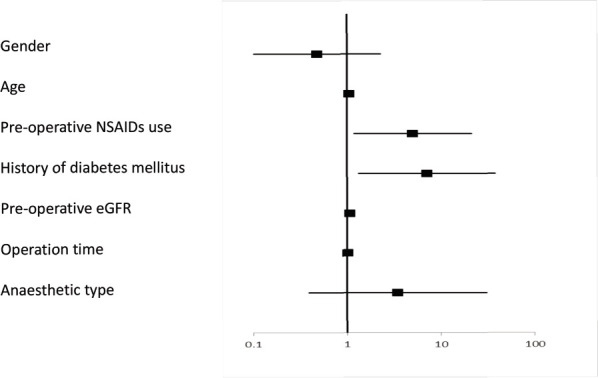
95% Confidence Interval of factors associated with deterioration of renal function one week after THA.

The mean pre-operative eGFR was 76.5ml//min/1.73m^2^ (standard deviation (SD) ±18.0, range 38.1-142.4). Prior to THA, 20% of all patients met the CKD criterion of eGFR <60ml/min/1.73m^2^ (eGFR categories G3a-G5). Univariate analysis showed that age at THA and use of pre-operative diuretics were significant risk factors for CKD (p<0.001 and p<0.001, respectively) (Table II). A significant correlation was found between age at THA and eGFR before THA (R=-0.420, p<0.001). The elderly showed significantly lower preoperative renal function (Fig. 2). In the multivariate logistic regression analysis, age at THA and use of pre-operative diuretics remained significant risk factors for CKD (OR 1.114, p<0.001; and OR 6.902, p=0.031, respectively) (Table II, Fig. 3). These patients were not excluded from the original cohort used for investigation of AKI in this study. Seven days after THA, the mean pre-operative eGFR was 79.2ml/min/1.73m^2^ (SD ±17.9, range 35.2-125.0). At the same point in time, the overall incidence rate of AKI was 0.49% (n=1). The patient who was diagnosed with AKI was an 84-year-old female. Her BMI was 21.4kg/m^2^ and she had a history of DM. She had not taken any NSAIDs, but did use ARBs before THA. Fourteen (6.9%) out of the 203 patients were classified into the “deterioration of the renal function group” seven days after THA according to this study’s criteria. The mean pre-operative eGFR in those 14 patients was 74.7ml/min/1.73m^2^ (SD ±23.0, range 43.3-115.4). On the other hand, the mean pre-operative eGFR in the 189 patients that did not meet these criteria was 79.5ml/min/1.73m^2^ (SD ±17.4, range 35.2-125.0). In the univariate analysis, pre-operative oral administration of NSAIDs, history of DM, and general anesthesia were determined as significant risk factors for deterioration of renal function (p=0.046, p=0.015, and p=0.039, respectively) (Table III). In the multivariate logistic regression analysis, the pre-operative oral administration of NSAIDs, history of DM, and high pre-operative eGFR were significant risk factors for deterioration of renal function (OR 5.014, p=0.028; OR 7.106, p=0.022; and OR 3.456, p=0.001, respectively) (Table III, Fig. 4).

*CKD, chronic kidney disease; ACEi, angiotensin-converting enzyme inhibitor; ARB, angiotensin-II receptor blocker

*THA, total hip arthroplasty; NSAIDs, nonsteroidal anti-inflammatory drugs; eGFR, estimated glemerular filtration rate.

**Table II:** Factors associated with pre-operative CKD

Descriptor	Pre-operative CKD N=40	No Pre-operative CKD N=163	Univariate P value	Multivariate Odds Ratio	95% Confidence Interval	P value
Gender (Male, Female)	10, 30	24, 139	0.119	2.424	0.877-6.705	0.088
Age (years) (mean±SD, range)	73.9±9.7, 43-90	63.2±10.1, 37-87	<0.001	1.114	1.065-1.165	<0.001
BMI (kg/m^2^) (mean±SD, range)	24.5±3.7, 17.7-34.7	24.2±4.2, 16.0-42.7	0.736			
Pre-operative NSAIDs use						
Yes	12 (30%)	43 (26%)	0.644			
No	28 (70%)	120 (74%)				
Pre-operative ACEi/ARB use						
Yes	14 (35%)	36 (28%)	0.089	1.197	0.494-2.897	0.690
No	26 (65%)	127 (78%)				
Pre-operative diuretics use						
Yes	6 (15%)	2 (1%)	<0.001	6.902	1.191-40.006	0.031
No	34 (85%)	161 (99%)				
Pre-operative statin use						
Yes	12 (30%)	27 (17%)	0.053	1.363	0.538-3.455	0.514
No	28 (70%)	136 (83%)				
History of diabetes mellitus						
Yes	7 (18%)	13 (8%)	0.07	0.973	0.277-3.418	0.966
No	33 (82%)	150 (92%)				

*CKD, chronic kidney disease; SD, standard deviation; BMI, body mass index; NSAIDs, nonsteroidal anti-inflammatory drugs; ACEi, angiotensin-converting enzyme inhibitor; ARB, angiotensin-II receptor blocker.

**Table III:** Factors associated with deterioration of renal function one week after THA

Descriptor	Deterioration of renal function N=14	No deterioration of renal function N=189	Univariate P value	Multivariate Odds Ratio	95% Confidence Interval	P value
Gender (Male, Female)	4, 10	30, 159	0.22	0.479	0.102-2.259	0.352
Age (years) (mean±SD, range)	69.2±8.1, 54-84	65.0±11.0, 37-90	0.164	1.054	0.991-1.121	0.097
BMI (kg/m^2^)	24.6±4.1,	24.3±4.1,				
(mean±SD, range)	16.8-30.5	16.0-42.7	0.748			
Pre-operative NSAIDs use						
Yes	7 (50%)	48 (25%)	0.046	5.014	1.187-21.174	0.028
No	7 (50%)	141 (75%)				
Pre-operative ACEi/ARB use						
Yes	5 (36%)	45 (24%)	0.318			
No	9 (64%)	144 (76%)				
Pre-operative diuretics use						
Yes	1 (7%)	7 (4%)	0.523			
No	13 (93%)	182 (96%)				
Pre-operative statin use						
Yes	3 (21%)	36 (19%)	0.827			
No	11 (79%)	153 (81%)				
History of diabetes mellitus						
Yes	4 (29%)	16 (8%)	0.015	7.106	1.332-37.916	0.022
No	10 (71%)	173 (92%)				
Pre-operative eGFR	89.8±26.1,	89.8±9.7, 43-90	0.07	1.057	1.023-1.093	0.001
	53.3-142.4					
Post-operative NSAIDs use						
Yes	14 (100%)	180 (95%)	0.404			
No	0 (0%)	9 (5%)				
Operation time (min)	56.1±25.0, 30-120	48.7±9.7, 43-90	0.162	1.007	0.984-1.031	0.543
(mean±SD, range)						
Total blood loss (g)	287±143, 617-77	293±9.7, 43-90	0.885			
(mean±SD, range)						
Anaesthetic type						
Spinal	12 (86%)	183 (97%)	0.039	3.456	0.390-30.654	0.265
General	2 (14%)	6 (3%)				

*THA, total hip arthroplasty; SD, standard deviation; BMI, body mass index; NSAIDs, nonsteroidal anti-inflammatory drugs; ACEi, angiotensin-converting enzyme inhibitor; ARB, angiotensin-II receptor blocker; eGFR, estimated glemerular filtration rate.

## Discussion

Acute deterioration of kidney function is a sudden and sustained decrease in renal function resulting in retention of urea, creatinine, and other waste products^5^. Indeed, 12% of patients do not experience normalisation of kidney function^21^. The occurrence of acute deterioration of kidney function and requirement of renal replacement therapy increase morbidity, mortality, hospital bed use, and costs^22^. Therefore, determining predictors of acute renal function decline in patients who undergo THA would reduce its frequency and the need for renal replacement therapy. In other words, it would reduce the morbidity associated with renal replacement therapy, mortality, hospital utilisation, and associated costs, and help physicians prepare for this catastrophic complication in advance.

Some definitions of acute deterioration of kidney function, including AKI, employed in clinical studies are extremely complex with graded increments in serum creatinine for different baseline serum creatinine values^23, 24^. The lack of consensus on the quantitative definition of acute deterioration of kidney function hinders clinical research in particular, since it confounds comparisons between studies. The RIFLE criteria were presented as the first systematic diagnostic criteria in 2004. In the current study, we used the updated, widely accepted RIFLE definition of AKI and CKD^20^, which allowed comparison with other patient groups, including both orthopaedic and other patients. For example, post-operative AKI after cardiovascular surgery can be compared with post-operative AKI after orthopaedic surgery.

This study showed that the incidence rate of AKI after THA was 0.49%, which is substantially lower than that in previous reports^10-12, 15^. This discrepancy could be attributed to varying predisposition to AKI among different ethnic groups^9, 25^. Notably, this study is the first to report the incidence of AKI after THA in a Japanese population. Moreover, the difference in rates may also be due to the lower BMI of the Japanese population than that of Western populations. High BMI is associated with increased risk of complications, including AKI. In Japan, a BMI ≥25 is generally used to define obesity. According to a survey conducted in 2001, the rate of adult obesity (BMI ≥25) in Japan was 28.0% among men (2.9% for BMI ≥30) and 21.6% among women (3.4% for BMI≥30), and the proportion of those with BMI≥30 was only approximately 1/10 of that in the United States^26^.

In addition, a variety of other factors may be associated with the lower incidence of AKI in Japanese populations, including differences in the incidence of lifestyle diseases such as DM and hypertension, and the amount of analgesics used. For example, in 2015, the number of Japanese people with DM was estimated to be about 7.2 million, the ninth highest in the world. However, the estimates in China, India, and the United States are 109.6 million, 69.2 million, and 29.3 million, with the third largest country, the United States, having rates four times those of Japan^27^.

Moreover, the types and doses of analgesics used in each country and institution differ, making direct comparisons difficult. Globally, acetaminophen is listed as the first choice analgesic in several guidelines, but in Japan, NSAIDs are generally the first choice^28, 29^. This difference between Japan and the West may also contribute to the difference in the incidence of AKI after THA.

Although the incidence rate of AKI after THA was low, acute deterioration of kidney function occurred in some patients. Thus, in the current study, we investigated patients with post-operative eGFR ≥10 ml/min/1.73 m^2^.

The number of post-THA AKI cases that met the RIFLE criteria was small, and we believed that it would be insufficient to appropriately identify risk factors. The SD of the change in eGFR from pre-operative to post-operative values was 8.49ml/min/1.73m^2^. Therefore, in this study, we used an eGFR change of 10ml/min/1.73m^2^ as our own diagnostic criterion. Thus, we have used different criteria to assess deterioration of renal function that does not meet the RIFLE criteria, and examined the risk factors in these cases. The prevalence of CKD generally increases with age^30^. In the current study, a significant correlation was found between eGFR and CKD. Pre-operative renal dysfunction has been reported as a risk factor for post-operative complications, including AKI and cardiovascular disease, which leads to prolonged morbidity or hospitalisation, and increased mortality^14^. Pre-operative renal dysfunction was not a risk factor for AKI following THA in this study; however, since a high percentage of patients met the criteria for CKD prior to THA, careful observation of renal function in the peri-operative period is required.

Insulin resistance results in widespread endothelial dysfunction and impaired renovascular autoregulation, as well as worse kidney injury following ischemia, as shown in murine models. Therefore, DM is a well-known risk factor for deterioration of renal function. In a previous report, patients with DM were 1.3 (for those on oral therapy) to 1.7 (for those on insulin therapy) times more likely to develop post-operative AKI^31^. The degree of increased risk in acute deterioration of kidney function according to our multivariate regression model was 7.1. However, the precise association between DM and AKI after THA is unclear.

NSAIDs affect renal autoregulation through impaired synthesis of renal prostaglandins, which reduces renal plasma flow and GFR^13^. Although use of NSAIDs is widely known to disrupt renal function, its correlation with post-joint arthroplasty kidney dysfunction is still under debate^11^. However, pre-operative use of NSAIDs was significantly correlated with AKI in previous reports^9, 11, 13^, and showed a high odds ratio of 5.0 (p=0.028) of acute deterioration of kidney function in the current study. In surgical patients with normal renal function, a transient decrease in function is observed but is considered to be clinically irrelevant. In the setting of stressed kidneys, continued NSAIDs administration should be avoided. Nevertheless, whether withholding NSAIDs from patients at higher risk of injury would affect the rate or magnitude of AKI is unknown^13^.

In this study, renal function was significantly reduced after THA in patients with high eGFR prior to surgery. This indicates that renal function deteriorated renal after THA. CKD was previously identified as a risk factor for AKI in patients undergoing THA^10, 32^, an association that was not observed in the current study. However, our study group was defined by a reduction in eGFR of ≥10ml/min/1.73m^2^ seven days following THA, which may have contributed to this significant discrepancy.

In the current study, advanced age; use of ACEIs/ARBs, diuretics, or statins; operation time; total blood loss; type of anesthetic (general anesthesia); and BMI were not significantly associated with risk of acute deterioration of kidney function. These factors were reported as risk factors for AKI in some studies^10-14, 16, 31, 33^, but not others^10, 11, 13, 14, 16^. The discrepant findings may be due to differences in ethnicity and prevalence, among others.

Other factors that were reported in previous reports include advanced age, use of antibiotics, revision surgery, and blood transfusion^9-12^. In patients with acute deterioration of kidney function, advanced age was identified as a significant risk factor for complications after total joint arthroplasty^12^. In line with previous reports, advanced age and pre-operative kidney dysfunction were also significantly associated with AKI^10^. In particular, patients older than 85 years were more likely to have severe AKI^31^. In the current study, advanced age was an independent significant risk factor for preoperative kidney dysfunction. However, no significant relationship was noted between advanced age and acute deterioration of kidney function after THA. This result may be due to the smaller age variation in this study than in other studies^9, 13, 14^. Age was shown to be a non-modifiable marker in the peri-operative context in many studies; thus, sufficient attention from medical staff is required.

None of the patients in this study received blood transfusion; thus, this factor’s influence could not be investigated. Because surgical invasiveness strongly influences AKI^11^, revision THA is speculated to pose a higher risk of AKI than primary THA. In addition, blood transfusion rates also increase with revision THA, which further increases the risk of AKI. All patients in this study were undergoing primary THA; nevertheless, paying sufficient attention to AKI and renal function deterioration before revision THA is necessary.

Antibiotics, aminoglycosides, and vancomycin were reported as significant predictors of AKI^11, 16, 34^. Patients receiving vancomycin and cefazolin as prophylactic antibiotics were more likely to develop AKI compared with those receiving only cefazolin^11, 34^. Use of vancomycin with cefazolin also results in a greater severity of kidney injury based on the Acute Kidney Injury Network (AKIN) criteria. However, the mechanism linking vancomycin to development of AKI has not been elucidated.

Although important advances have been made in improving our understanding of the pathophysiology and, in particular, recovery from peri-operative renal injury, no effective therapy has been found. No drug has been proven to decrease the incidence of peri-operative renal failure.

Collaboration between surgeons and internists to limit NSAIDs use and strictly control blood glucose should be considered as a preventive measure to reduce AKI following THA. For example, oral hypoglycemic agents or insulin can be used prior to surgery while monitoring HbA1c levels, and other drugs such as acetaminophen should be pre-operatively administered in place of NSAIDs. Therefore, DM and the use of NSAIDs can be classified as controllable risks, and a multidisciplinary approach would be reasonable to minimise peri-operative AKI or acute deterioration of kidney function. Thus, a carefully planned approach incorporating interdisciplinary peri-operative care is warranted to reduce both the risk and the consequences of this potentially devastating condition.

This study has several limitations, which are inherent to all retrospective studies. First, the study group was relatively small. Previous studies suggested that older age, higher BMI, CKD, and peri-operative blood transfusion were risk factors for AKI following THA^17^; however older age, higher BMI, CKD, and total blood loss were not significantly associated with AKI following THA in this study. This may be due to the small number of patients in this study, and future studies with larger cohorts are necessary. Second, the significance of urine volume could not be evaluated because post-operative urine output was not measured. Third, evaluating the effect of intravenous infusion on renal function is difficult, although it is used after THA to prevent intravascular dehydration. Fourth, the volume of intravenous hydration and the severity of dehydration could not be assessed in this study. Whether kidney function had improved after the first week of THA or not is unknown in patients with permanent deterioration of renal function. In addition, whether exacerbation of renal function was temporary and whether the deterioration of renal function was clinically significant are also unclear. Future double-blind randomised control trials on the pre-operative use of NSAIDs could provide useful information with respect to their effects on renal function. In addition, prospective long-term studies involving a larger number of patients are needed to further substantiate our findings.

## Conclusion

We conducted a retrospective cohort study about the risk factors for AKI or acute deterioration of kidney function after primary THA. This study showed that the incidence rates of AKI and acute deterioration of kidney function, which may occur around the time of primary THA, were 0.49% and 6.9%, respectively. DM and NSAIDs use were controllable risks, and a multidisciplinary approach is reasonable to minimise peri-operative acute kidney injury or acute deterioration of kidney function. While DM and preoperative NSAIDs use cannot be controlled during the peri-operative period, they can be controlled through patient management prior to surgery. Limitation of NSAIDs use and strict control of blood glucose in conjunction with HbA1c level monitoring should be considered preventive measures for reduction of AKI following THA. We confirmed that diligent medical management not only during the peri-operative period but also prior to surgery is necessary for patients undergoing THA.
